# Normal B cell development and Pax5 expression in *Thy28/ThyN1*-deficient mice

**DOI:** 10.1371/journal.pone.0220199

**Published:** 2019-07-22

**Authors:** Fusako Kitaura, Miyuki Yuno, Toshitsugu Fujita, Shigeharu Wakana, Jun Ueda, Kazuo Yamagata, Hodaka Fujii

**Affiliations:** 1 Chromatin Biochemistry Research Group, Combined Program on Microbiology and Immunology, Research Institute for Microbial Diseases, Osaka University, Yamadaoka, Suita, Osaka, Japan; 2 Department of Biochemistry and Genome Biology, Hirosaki University Graduate School of Medicine, Zaifu-cho, Hirosaki, Aomori, Japan; 3 Technology and Development Team for Mouse Phenotype Analysis, RIKEN BioResource Research Center, Koyadai, Tsukuba, Ibaraki, Japan; 4 Department of Gerontology, Institute of Biomedical Research and Innovation, Minatojima Minamiachi, Chuo-ku, Kobe, Japan; 5 Center for Genetic Analysis of Biological Responses, Research Institute for Microbial Diseases, Osaka University, Yamadaoka, Suita, Japan; 6 Center for Advanced Research and Education (CARE), Asahikawa Medical University, Asahikawa, Japan; 7 Faculty of Biology-Oriented Science and Technology, Kindai University, Nishimitani, Kinokawa City, Wakayama, Japan; Chang Gung University, TAIWAN

## Abstract

Thy28, also known as ThyN1, is a highly conserved nuclear protein. We previously showed that in a chicken mature B cell line, Thy28 binds to the promoter of the gene encoding *Pax5*, a transcription factor essential for B cell development, and positively regulates its expression. Here, we generated a *Thy28*-deficient mouse line to analyze its potential role in B cell development in mice. *Thy28*-deficient mice showed normal development of B cells, and the expression of Pax5 was comparable between wild-type and *Thy28*-deficient primary B cells. Thus, species-specific mechanisms regulate Pax5 expression and B cell development.

## Introduction

B cell development is a complex process regulated by the concerted actions of many gene products. Pax5 is an essential transcription factor in the process of B cell development [1]. Expression of the mouse *Pax5* gene is regulated by many transcription factors and DNA-binding proteins. Examples of such regulators include PU.1, IRF4, IRF8, NF-κB, and EBF1 [2, 3]. We previously used a locus-specific chromatin immunoprecipitation (ChIP) approach to analyze the mechanisms regulating the expression of *Pax5* in a chicken mature B cell line, DT40 [4][5]. We found that Thy28, which is also known as ThyN1, binds to the promoter region of the *Pax5* gene in a B cell-specific manner and positively regulates its expression [6].

Thy28 is an evolutionarily-conserved protein [7, 8] that is highly expressed in the bursa of Fabricius and in other lymphoid tissues in the chicken [7]. It is also expressed in the liver, heart, and brain in chickens [7]. In contrast to its relatively limited tissue distribution in the chicken, Thy28 is more broadly expressed in the mouse [8].

In the present study, we generated a mutant mouse strain lacking expression of Thy28 to examine its *in vivo* function in mice. The *Thy28*-deficient (Thy28^-/-^) mice were viable and showed normal development. Interestingly, B cell development in Thy28^-/-^ mice was normal, suggesting that Thy28 is dispensable for B cell development in mice. Expression of Pax5 was comparable between wild-type and Thy28^-/-^ primary B cells. These results suggest a species-specific role of Thy28 in B cell development and function.

## Materials and methods

### Mice

The targeting vector for the mouse *Thy28* gene (PG00147_X_4_A07) was obtained from the European Conditional Mouse Mutagenesis Program (EUCOMM). The linearized plasmid was transfected into an embryonic stem (ES) cell line, EGR-G101, which was previously established from C57BL/6-Tg(CAG/Acr-EGFP)C3-N01-FJ002Osb mice, as described previously [9]. After G418 selection, surviving colonies were subjected to screening by PCR. ES cells retaining the transgene in the *Thy28* locus were injected into blastocysts derived from ICR mice (Japan SLC) to generate chimeras. The chimeric mice were crossed with C57BL/6 mice to generate heterozygous Thy28^KI/+^ mice (strain name: C57BL/6-Thyn1^*tm1a(EUCOMM)Osb/Osb*^) (RIKEN BioResource Center RBRC09564). The Thy28^KI/+^ mice were then crossed with CAG-FLPe mice [10] to generate Thy28^flox/+^ mice (strain name: B6.Cg-Thyn1^*tm1c(EUCOMM)Osb/Osb*^) (RIKEN BioResource Center RBRC09563), and the Thy28^flox/+^ mice were crossed with CAG-Cre mice [11] to generate Thy28^+/-^ mice (strain name: B6.Cg-Thyn1^*tm1d(EUCOMM)Osb/Osb*^) (RIKEN BioResource Center RBRC09565). Finally, the Thy28^+/-^ mice were crossed with each other to generate Thy28^+/^, Thy28^+/-^, and Thy28^-/-^ mice.

All animal experiments were approved by the Institutional Animal Care and Use Committee at the Research Institute for Microbial Diseases, Osaka University.

### Genotyping

For genotyping, genomic DNA was extracted and subjected to PCR with KOD FX (Toyobo). PCR conditions were as follows. Thy28^KI/+^ mice: heating at 94°C for 2 min, followed by 35 cycles of 98°C for 10 s, 68°C for 10 min, and 68°C for 2 min. Thy28^flox/+^ mice: heating at 94°C for 2 min, followed by 37 cycles of 94°C for 20 s, 64°C for 20 sec, 72°C for 30 sec, and 72°C for 10 min. Tny28^+/-^ mice: heating at 94°C for 2 min; followed by 35 cycles of 98°C for 10 s, 62°C for 30 sec, 68°C for 6 min, and 68°C for 2 min. Primers used for genotyping PCR are shown in Table 1.

**Table 1:** Oligodeoxyribonucleotides used in this study.

Number	Name	Sequence (5' → 3')	Experiments
27379	5’Gene-Specific (GF3)	gcaagtgtcaggccagtctgaggcaacatg	Genotyping of Thy28^KI/+^ mice
27246	LAR3+2	cctacatagttggcagtgtttggggcaagtg	Genotyping of Thy28^KI/+^ mice
26859	pNT1.1-Neo-R4	atggcgatgcctgcttgccgaatatcatgg	Genotyping of Thy28^KI/+^ mice
27250	3'Gene-Specific (GR4)	cgagaacgacacaatagcgaagtatgag	Genotyping of Thy28^KI/+^ mice
27701	LAR3+1_F	caacaagtttgtacaaaaaagcaggctggc	Genotyping of Thy28^floxed/+^ and Thy28^KI/+^ mice
27702	R2R_R	Ccgcctactgcgactataga	Genotyping of Thy28^floxed/+^ and Thy28^KI/+^ mice
27667	Thy28 Fow2	tatgtatccagccccaagaacagt	Genotyping of Thy28^+/-^ mice
27668	Thy28 Rev2	agggtgagactgaggtgtttatcg	Genotyping of Thy28^+/-^ mice

### Immunoblot analysis

Nuclear extracts (NE) were prepared with NE-PER Nuclear and Cytoplasmic Extraction Reagents (Thermo Fisher Scientific). Aliquots of NE (10 μg) were subjected to immunoblot analysis with an anti-Thy28 Ab (kindly gifted by Dr. Compton) [7], as described previously [12].

### Cell staining and flow cytometry

Cells were stained for 30 min at 4°C with fluorochrome-conjugated antibodies (Abs). Abs used for surface staining were fluorescein isothiocyanate (FITC)-conjugated mouse CD19 (130-102-494, Miltenyi), phycoerythrin (PE)-Cy7-conjugated mouse CD3 (552774, BD Bioscience), allophycocyanin (APC)-conjugated mouse IgD (405713, BioLegend), APC-Cy7-conjugated mouse MHC class II (107628, BioLegend), BV510-conjugated mouse CD19 (562956, BD Pharmingen), BV421-conjugated CD5 (562739, BD Pharmingen), and PE-conjugated CD21/35 (552957, BD Pharmingen).

For detection of Pax5 protein, splenocytes from 7-week-old mice were stained with FITC-labeled anti-CD19 in autoMACS Running Buffer—MACS Separation Buffer (130-091-221, Miltenyi), followed by staining with a PE-conjugated anti-Pax5 Ab (12–9918, eBioscience/Thermo Fisher Scientific) according to the manufacture's protocol. Flow cytometric analysis was performed on a FACSCalibur (BD Biosciences) and data was analyzed with FlowJo software (TreeStar).

### Statistics

Prism 8 software (GraphPad) was used for statistical analyses. One-way analysis of variance (ANOVA) or Student t-tests were used to calculate p-values.

## Results and discussion

### Generation of Thy28^-/-^ mice

**Fig 1:**
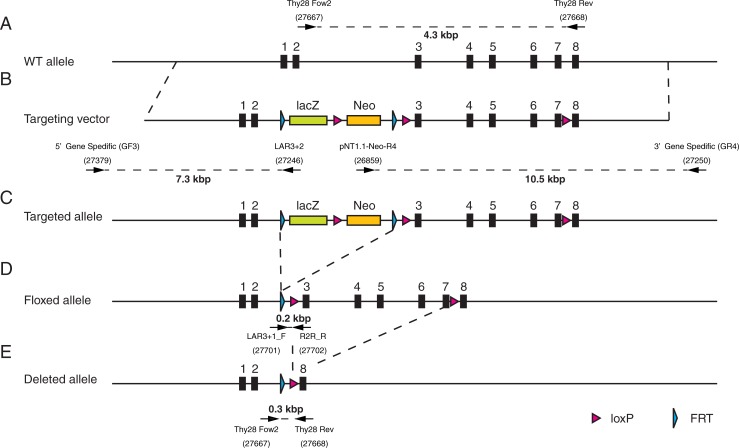
Generation of the *Thy28*-deficient mice. Schematic diagrams of the *Thy28* locus (**A**), the targeting vector (**B**), the targeted allele (**C**), the floxed allele (**D**), and the deleted allele (**E**).

**Fig 2:**
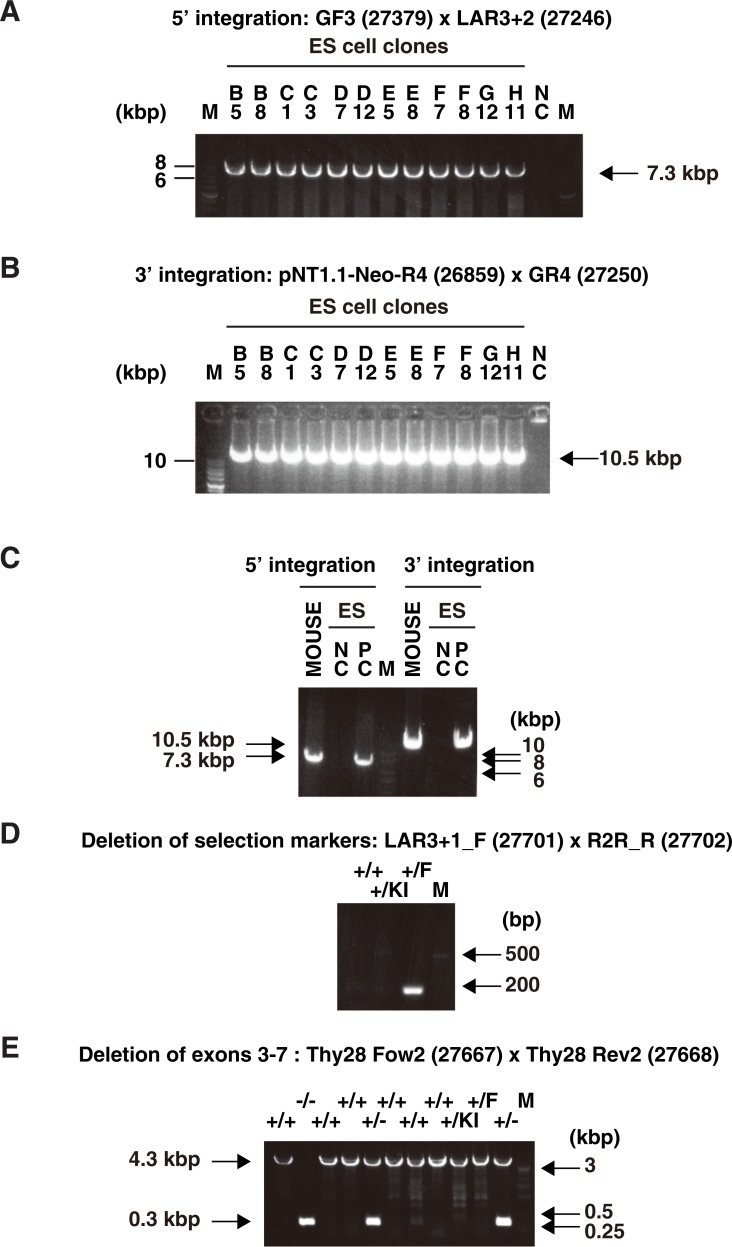
Genotyping of the *Thy28*-deficient mice. Detection of the 5' integration site (**A**) and the 3' integration site (**B**) in the ES cell clones. (**C**) Detection of 5' and 3' integration in an individual mouse. (**D**) Confirmation of deletion of selection markers by FLPe-mediated FRT recombination. (**E**) Confirmation of deletion of exons 3–7 of the *Thy28* gene by Cre-mediated loxP recombination.

**Fig 3:**
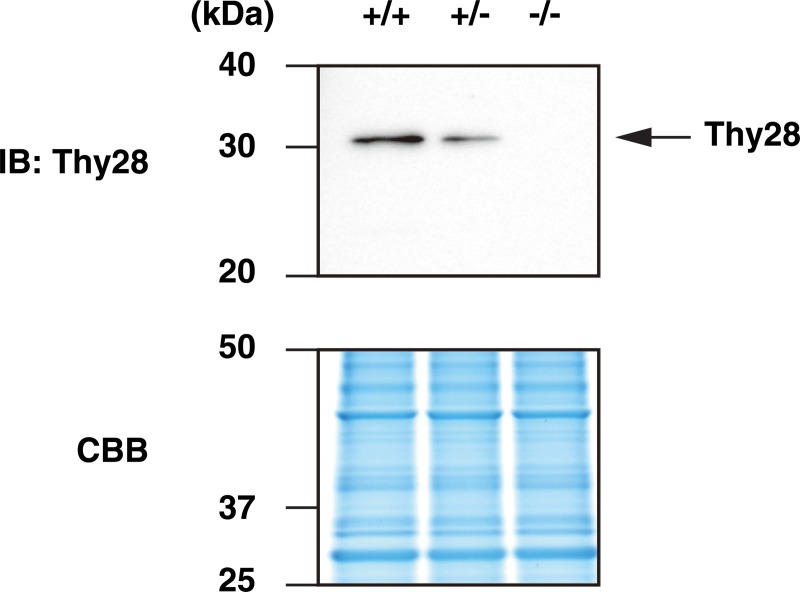
Expression of Thy28 in Thy28-mutant mice. Expression of Thy28 in murine spleocytes was detected by immunoblot analysis with an anti-Thy28 Ab. Coomassie Brilliant Blue (CBB) staining is shown as a protein loading control.

To examine the potential role of Thy28 in B cell development in mice, we generated mutant mice in which the *Thy28* gene was inactivated by deletion of its exons 3–7 (Thy28^-/-^ mice) (Figs 1 and 2, Table 1). The linearized targeting vector for the mouse *Thy28* gene was transfected into an ES cell line, EGR-G101 [9]. After G418 selection, surviving colonies were subjected to screening by PCR. ES cells retaining the transgene in the *Thy28* locus were injected into blastocysts derived from ICR mice to generate chimeras. The chimeric mice were crossed with C57BL/6 mice to generate heterozygous Thy28^KI/+^ mice. The Thy28^KI/+^ mice were crossed with CAG-FLPe mice [10] to generate Thy28^flox/+^ mice, and the Thy28^flox/+^ mice were crossed with CAG-Cre mice [11] to generate Thy28^+/-^ mice. Finally, the Thy28^+/-^ mice were crossed each other to generate Thy28^+/^, Thy28^+/-^, and Thy28^-/-^ mice. The Thy28^-/-^ mice were viable and born in the expected Mendelian ratios (Table 2), suggesting that the *Thy28* gene is dispensable for normal development. As expected, the expression of Thy28 protein was lost in Thy28^-/-^ mice, and reduced in heterozygous Thy28^+/-^ mice (Fig 3). These results indicated that our targeting strategy effectively knocked out the *Thy28* gene in these mice.

**Table 2:** Birth ratios of *Thy28*-deficient mice.

Number (%)	+/+	+/-	-/-
Male	35 (26.5%)	61 (46.2%)	36 (27.3%)
Female	30 (23.1%)	68 (52.3%)	32 (24.6%)
Total	65 (24.8%)	129 (49.2%)	68 (26.0%)

### Normal development of B cells in Thy28^-/-^ mice

**Fig 4:**
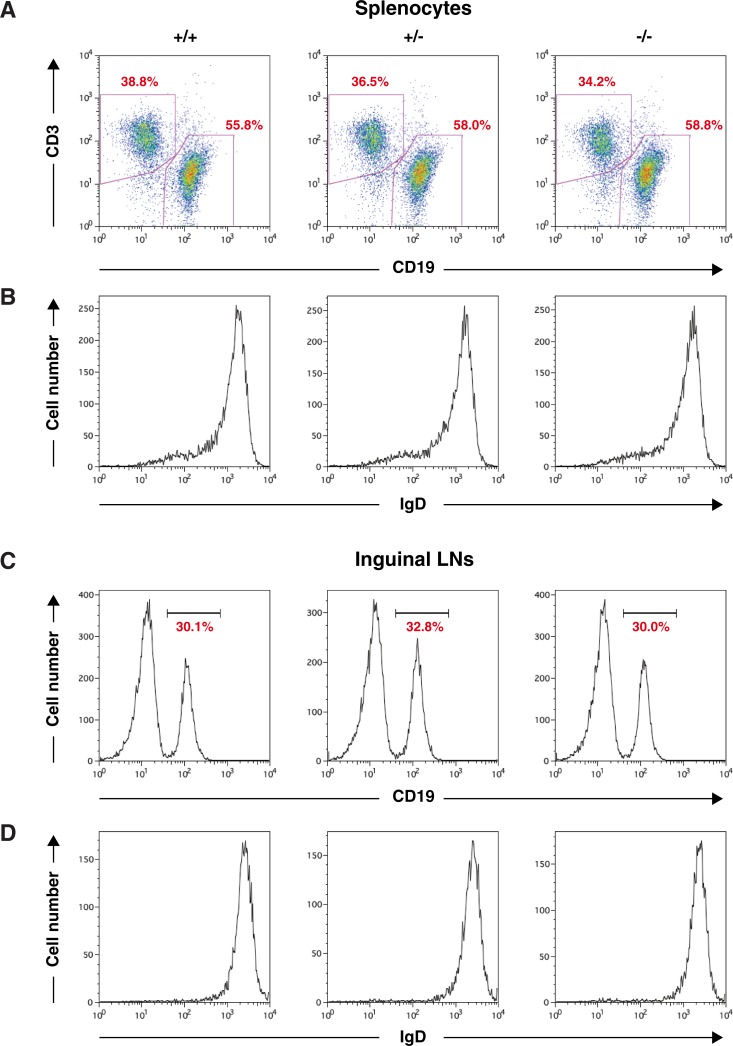
B cell profiles. (**A**) Percentages of B cells and T cells in the spleen. (**B**) Expression of IgD on splenic B cells. (**C**) Percentages of CD19^+^ B cells in the inguinal lymph nodes (LNs). (**D**) Expression of IgD on B cells in the inguinal LNs.

**Fig 5:**
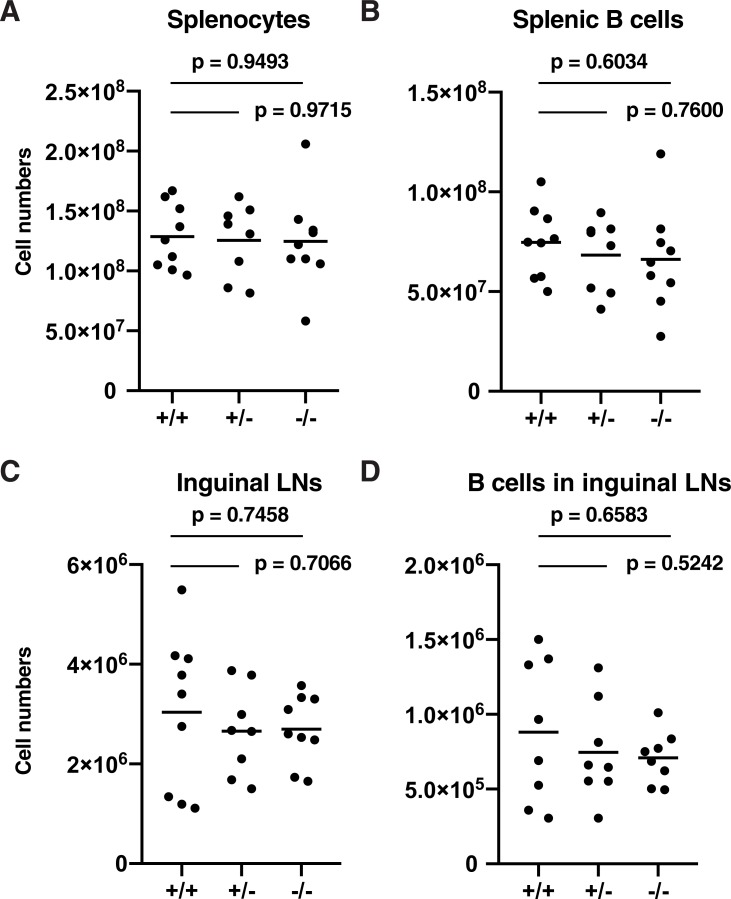
Numbers of B cells in spleens and inguinal LNs. Numbers of (**A**) splenocytes, (**B**) splenic CD19^+^ B cells, (**C**) cells in inguinal LNs, and (**D**) CD19^+^ B cells in inguinal LNs are shown. The age range of the mice was 8.1–38.7 weeks. One-way ANOVA was used to calculate p-values.

**Fig 6:**
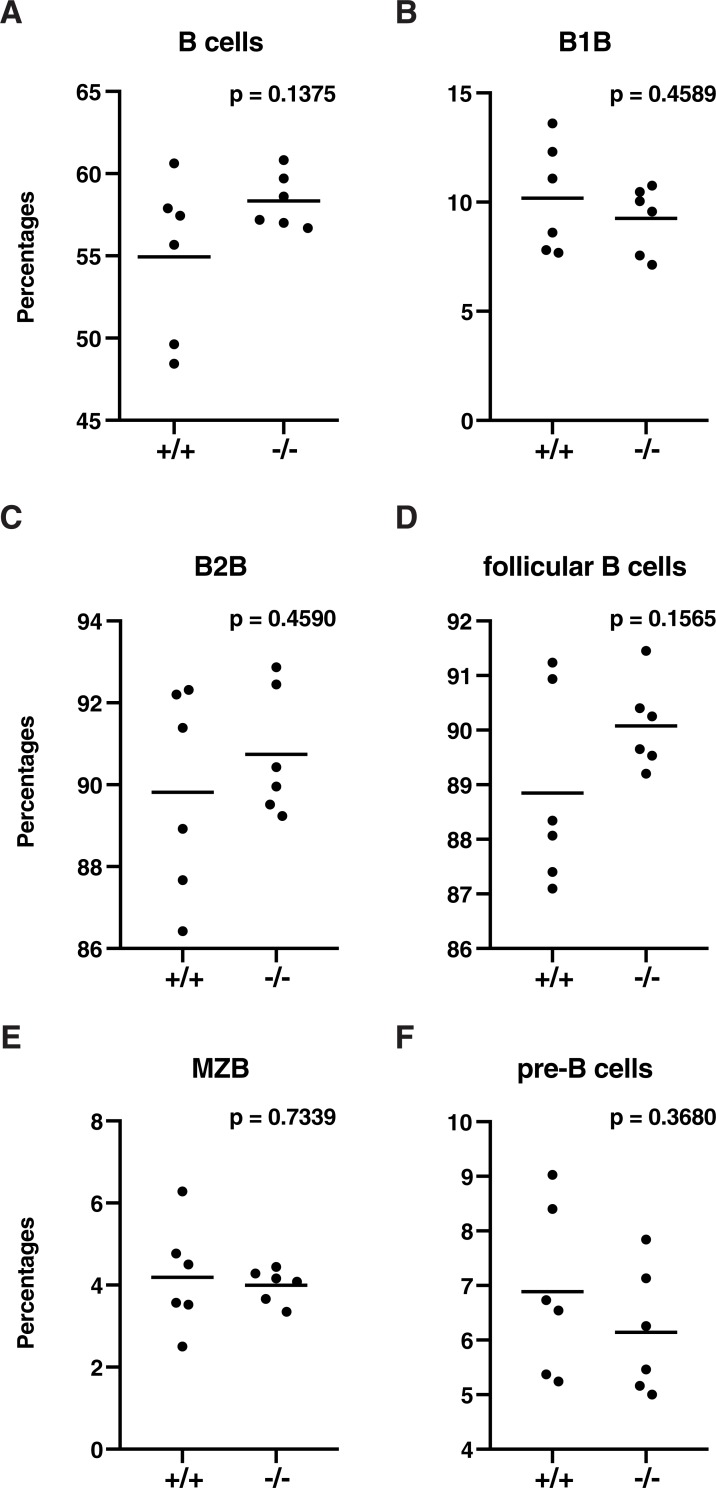
Cellularity of B cells in the spleen. (**A**) B cells, (**B**) B1B cells, (**C**) B2B cells, (**D**) follicular B cells, (**E**) marginal zone B (MZB) cells, and (**F**) pre-B cells. Gating steps are as follows: B cells: MHC class II^+^ CD19^+^; B1B cells: MHC class II^+^ CD19^+^ CD5^+^; B2B cells: MHC class II^+^ CD19^+^ CD5^-^; follicular B cells: MHC class II^+^ CD19^+^ CD5^-^ CD21/35^+^; MZB cells: MHC class II^+^ CD19^+^ CD5^-^ CD21/35^high^; and pre-B cells: MHC class II^+^ CD19^+^ CD5^-^ CD21/35^low^. Student's t-tests were used to calculate p-values.

To examine the potential role of Thy28 in the development of mouse B cells and other lymphocytes, we analyzed the B cell population in Thy28^-/-^ mice. As shown in Figs 4 and 5, no abnormalities were detected in cellularity in the B cell population. B cell numbers in Thy28^-/-^ mice were normal, as determined by the percentages of CD19^+^ cells in the spleen and lymph node (LN) (Figs 4 and 5). The percentages of total B cells, B1B cells, B2B cells, follicular B cells, marginal zone B (MZB) cells, and pre-B cells in spleens from Thy28^-/-^ mice were normal (Fig 6). These data suggest that Thy28 is dispensable for B cell development in mice.

### Normal expression of Pax5 in Thy28^-/-^ B cells

**Fig 7:**
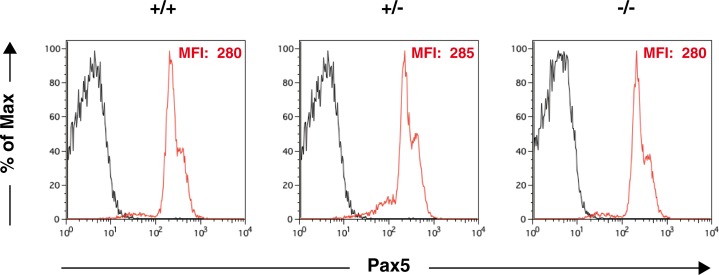
Expression of Pax5 in splenic B cells. Splenocytes from 7-week-old mice were stained with a FITC-conjugated anti-CD19 Ab and a PE-conjugated anti-Pax5 Ab. The expression level of Pax5 in CD19^+^ B cells is shown. The mean fluorescence intensity (MFI) of Pax5 staining is shown. Black: unstained control; red: Pax5 staining. Percentages of Pax5^+^ cells in CD19^+^ splenic B cells from Thy28^+/^, Thy28^+/-^, and Thy28^-/-^ mice were 95.6%, 94.7%, and 95.6%, respectively.

Finally, we examined the effect of the loss of Thy28 on the expression of Pax5. Expression of Pax5 in CD19^+^ splenic B cells was comparable between Thy28^+/^, Thy28^+/-^, and Thy28^-/-^ mice (Fig 7). Expression of Pax5 in mature B cells in inguinal LNs was also comparable between Thy28^+/^, Thy28^+/-^, and Thy28^-/-^ mice (S1 Fig). These data show that Thy28 is dispensable for Pax5 expression in mature B cells in the mouse. We previously showed that Thy28 binds to the promoter region of the *Pax5* gene in a B cell-specific manner in a chicken mature B cell line, DT40, and down-regulation of Thy28 resulted in a decrease in the expression of the *Pax5* gene [6]. These results in a chicken B cell line were in clear contrast with the present results in mice. We also knocked down Thy28 in the human B cell lines Nalm-6 and Raji. As shown in S2 Fig, down-regulation of Thy28 in these cell lines did not affect the expression of Pax5. These results demonstrate that Thy28 is dispensable for Pax5 expression in B cells from at least two mammals, mice and humans, and suggest a species-specific mechanism for the regulation of Pax5 expression.

## Conclusions

We generated *Thy28*-deficient mice to investigate the potential role of Thy28/ThyN1 in B cell development. *Thy28*-deficient mice were viable and showed a Mendelian birth ratio. *Thy28*-deficient mice had normal B cell numbers as well as normal percentages of subclasses of B cell lineages. Finally, the expression of Pax5 was normal in B cells from *Thy28*-deficient mice. These results indicate that Thy28/ThyN1 is dispensable for the regulation of Pax5 expression and the development of B cells in the mouse and suggest a species-specific role of Thy28/ThyN1 in Pax5 expression and B cell development.

## Supporting information

S1 Fig Expression of Pax5 in mature B cells in inguinal lymph nodes. Splenocytes from 9-week-old mice were stained with a FITC-conjugated anti-CD19 Ab, an APC-conjugated IgD Ab, and a PE-conjugated anti-Pax5 Ab. The expression of Pax5 in CD19^high^ and IgD^+^ B cells is shown. The mean fluorescence intensity (MFI) of Pax5 staining is shown. Percentages of Pax5^+^ cells in CD19^high^ and IgD^+^ B cells from Thy28^+/^, Thy28^+/-^, and Thy28^-/-^ mice were 99.6%, 99.7%, and 99.9%, respectively.
(PDF)

S2 Fig Expression of Pax5 in human B cell lines. (**A**, **B**) shRNA-mediated knock-down of Thy28 in a human pre-B cell line, Nalm-6. Expression of Pax5 protein (A) and *Pax5* mRNA (B) was analyzed in Nalm-6 cells stably expressing an shRNA against GFP or human Thy28. The expression of *Pax5* mRNA was quantified by real-time RT-PCR and normalized to the expression of *GAPDH* mRNA (mean +/- SEM, n = 4). (**C**, **D**) Clustered regularly interspersed short palindromic repeats (CRISPR)/CRISPR-associated protein 9 (Cas9)-mediated knock-out of Thy28 in a human Burkitt′s lymphoma cell line, Raji. (**C**) Nucleotide insertions or deletions generated by CRISPR/Cas9 in the human *Thy28* gene. The ATG codons in blue and the TGA codon in red indicate start codons and an inserted stop codon, respectively. The CRISPR/Cas9 target sequence is underlined. (**D**) Expression of Pax5 was analyzed in Thy28 mutant (KO) Raji cells.
(PDF)
