# Supplementary figures and images for: Normal B cell development and Pax5 expression in Thy28/ThyN1-deficient mice
Source: PLoS One. 2019 Jul 22;14(7):e0220199. doi: 10.1371/journal.pone.0220199 (PMC6645560; doi:10.1371/journal.pone.0220199)

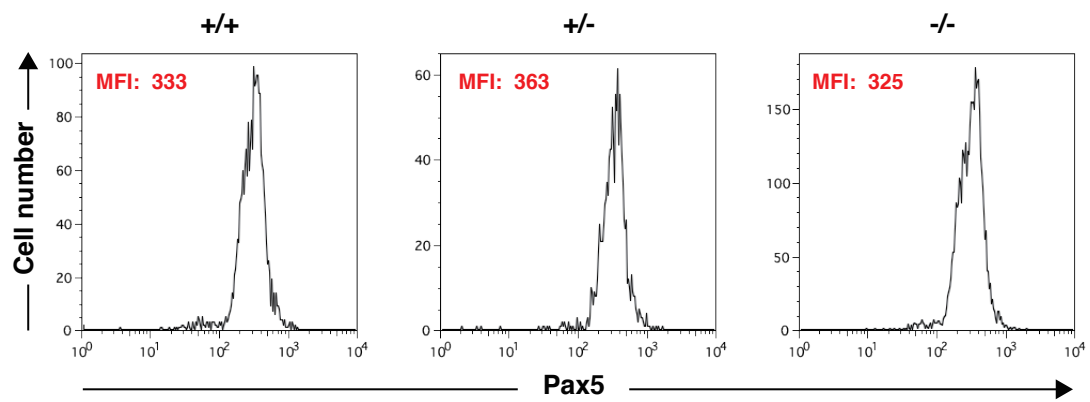

Supplement: S1 Fig — Splenocytes from 9-week-old mice were stained with a FITC-conjugated anti-CD19 Ab, an APC-conjugated IgD Ab, and a PE-conjugated anti-Pax5 Ab. The expression of Pax5 in CD19high and IgD+ B cells is shown. The mean fluorescence intensity (MFI) of Pax5 staining is shown. Percentages of Pax5+ cells in CD19high and IgD+ B cells from Thy28+/, Thy28+/-, and Thy28-/- mice were 99.6%, 99.7%, and 99.9%, respectively. (PDF) [file pone.0220199.s001.pdf]

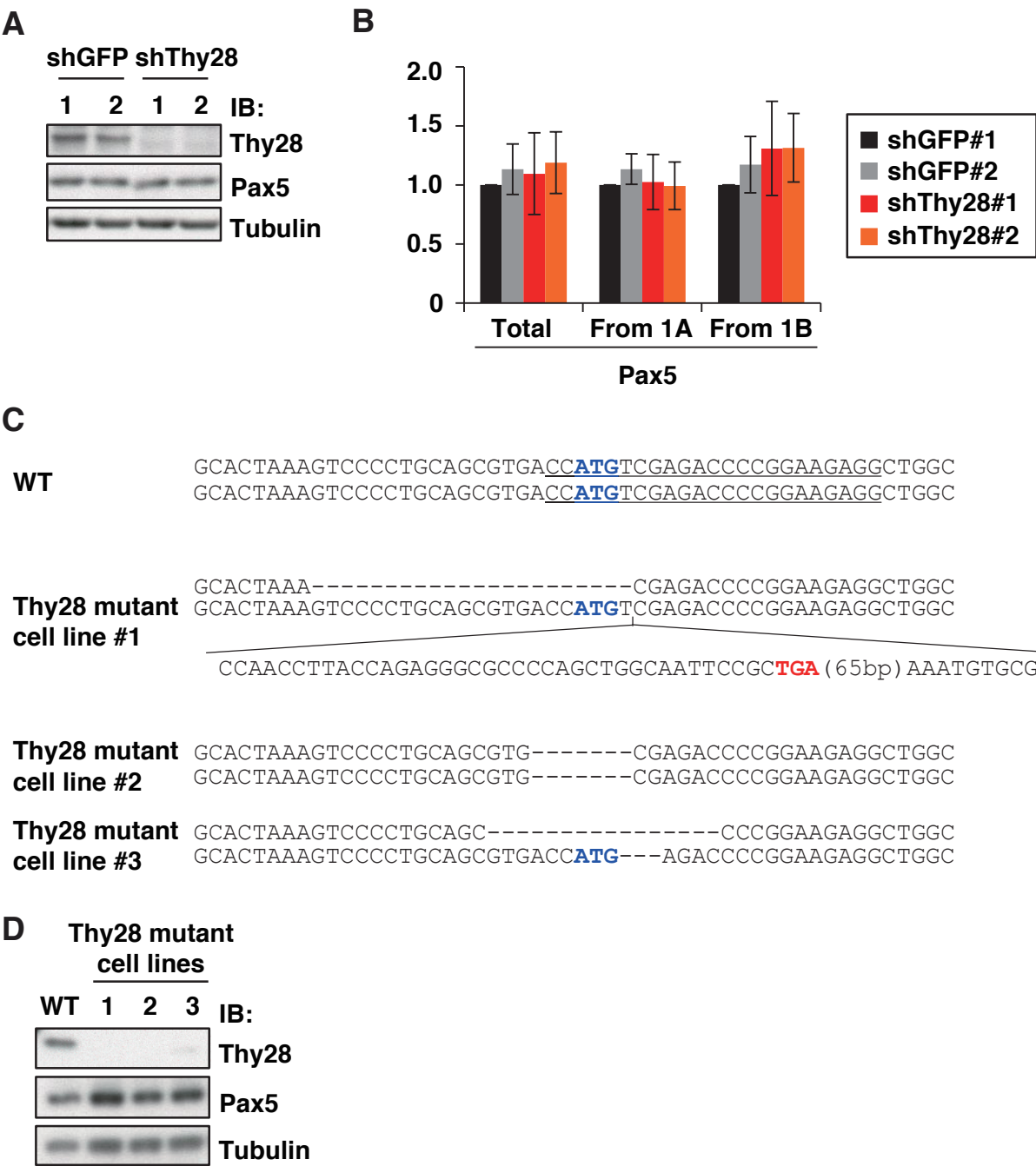

Supplement: S2 Fig — (A, B) shRNA-mediated knock-down of Thy28 in a human pre-B cell line, Nalm-6. Expression of Pax5 protein (A) and Pax5 mRNA (B) was analyzed in Nalm-6 cells stably expressing an shRNA against GFP or human Thy28. The expression of Pax5 mRNA was quantified by real-time RT-PCR and normalized to the expression of GAPDH mRNA (mean +/- SEM, n = 4). (C, D) Clustered regularly interspersed short palindromic repeats (CRISPR)/CRISPR-associated protein 9 (Cas9)-mediated knock-out of Thy28 in a human Burkitt′s lymphoma cell line, Raji. (C) Nucleotide insertions or deletions generated by CRISPR/Cas9 in the human Thy28 gene. The ATG codons in blue and the TGA codon in red indicate start codons and an inserted stop codon, respectively. The CRISPR/Cas9 target sequence is underlined. (D) Expression of Pax5 was analyzed in Thy28 mutant (KO) Raji cells. (PDF) [file pone.0220199.s002.pdf]
